# Foodborne Transmission of Bovine Spongiform Encephalopathy to Non-Human Primates Results in Preclinical Rapid-Onset Obesity

**DOI:** 10.1371/journal.pone.0104343

**Published:** 2014-08-04

**Authors:** Alexander Strom, Barbara Yutzy, Carina Kruip, Mark Ooms, Nanette C. Schloot, Michael Roden, Fraser W. Scott, Johannes Loewer, Edgar Holznagel

**Affiliations:** 1 Institute for Clinical Diabetology, German Diabetes Center, Leibniz Center for Diabetes Research, Düsseldorf, Germany; 2 Paul-Ehrlich-Institut, Federal Agency for Vaccines and Biomedicines, Langen, Germany; 3 Department of Endocrinology and Diabetology, Heinrich-Heine University, Düsseldorf, Germany; 4 Chronic Disease Program, Ottawa Hospital Research Institute, Ottawa, Ontario, Canada; The Scripps Research Institute Scripps Florida, United States of America

## Abstract

Obesity has become one of the largest public health challenges worldwide. Recently, certain bacterial and viral pathogens have been implicated in the pathogenesis of obesity. In the present study, we retrospectively analyzed clinical data, plasma samples and post-mortem tissue specimens derived from a risk assessment study in bovine spongiform encephalopathy (BSE)-infected female cynomolgus monkeys (*Macaca fascicularis*). The original study design aimed to determine minimal infectious doses after oral or intracerebral (i.c.) infection of macaques to assess the risk for humans. High-dose exposures resulted in 100% attack rates and a median incubation time of 4.7 years as described previously. Retrospective analyses of clinical data from high-dosed macaques revealed that foodborne BSE transmission caused rapid weight gain within 1.5 years post infection (β = 0.915; P<0.0001) which was not seen in age- and sex-matched control animals or i.c. infected animals. The rapid-onset obesity was not associated with impaired pancreatic islet function or glucose metabolism. In the early preclinical phase of oral transmission associated with body weight gain, prion accumulation was confined to the gastrointestinal tract. Intriguingly, immunohistochemical findings suggest that foodborne BSE transmission has a pathophysiological impact on gut endocrine cells which may explain rapid weight gain. To our knowledge, this is the first experimental model which clearly demonstrates that foodborne pathogens can induce obesity.

## Introduction

Obesity has been declared by the World Health Organization to be a global epidemic. To date, over one billion people worldwide are either overweight or obese. Behavioral, environmental, and genetic factors are considered to be the major contributors to obesity. However, over the last two decades evidence of pathogens as an obesity risk factor has accumulated [1]–[4].

Transmissible spongiform encephalopathies (TSEs) include bovine spongiform encephalopathy (BSE) in cattle [5] and variant Creutzfeldt-Jakob Disease (CJD) in humans [6]–[8]. Variant CJD was most likely caused by dietary exposure to BSE-contaminated food [9]. Experiments to investigate foodborne BSE transmission in cattle [10]–[12] and macaques [13], [14] confirmed the efficiency of the oral route. TSEs are characterized by the accumulation of a pathogenic, misfolded, aggregation prone isoform of the endogenous cellular prion protein (PrP^C^) [15]. One of the unique characteristics of the abnormal prion protein (PrP^Sc^) is its relative resistance to proteinase K-treatment resulting in a truncated molecule called PrP^res^ [16], [17]. PrP^C^ is expressed in a variety of tissues and cells [18]–[20] and is implicated in a wide range of cellular and physiological processes [21]–[26]. However, the exact physiological function of PrP^C^ is still unclear.

In rodents, intracerebral (i.c.) or intraperitoneal (i.p.) prion infections lead to metabolic disorders associated with disease onset. These disorders include obesity, hypoglycemia-hyperinsulinemia, and diabetes mellitus [27]–[29]. It was suggested that these metabolic abnormalities result from the damage of brain regions controlling central metabolic regulation. Recently, we described attack rates, incubation periods and prion spread in i.c. [30] and orally BSE-infected [13] cynomolgus monkeys (*Macaca fascicularis*). These studies are part of *in vivo* BSE titration studies to assess the risk for humans. To date, only studies in high-dosed macaques could be terminated. In the present retrospective study, we demonstrate that oral but not i.c. BSE infection resulted in rapid-onset obesity. The rapid weight gain was not associated with abnormal plasma glucose, insulin or glucagon levels. It also appeared long before the infectious agent could be detected in the CNS. Analysis of prion spread in preclinical orally infected macaques revealed that the prion load was restricted to the gastrointestinal (GI) tract during the period of body weight increase. Gut endocrine cells secrete incretin hormones which function partially as signals of satiety [31]. In a pilot study, we performed immunohistochemical analyses in the distal part of the ileum in order to get first insight whether oral prion transmission could have a pathophysiological impact on gut endocrine cells. Preliminary immunohistochemical data indicate reduced L-cell density in orally BSE-infected macaques that developed rapid onset obesity.

## Materials and Methods

The animal experiments were approved by the Hessian Animal Protection Committee (local authority permit no. V54–19c 20/15–F107/63, Regierungspräsdium Darmstadt), carried out in strict accordance with section 8 of the German Animal Protection Law and supervised by local authorities (Regierungspräsidium Offenbach).

All macaques were purchased as healthy female 1-year-old (n = 24) or as healthy male 3-year-old animals (n = 4) from the Centre de Recherche en Primatologie, Mauritius. All female macaques were randomly assigned to different animal groups with six animals each. Brain homogenates from U.K. BSE cattle were prepared and used for infection studies as described [13], [30]. Animals of group A were inoculated i.c. with 5 mg BSE brain homogenate each at the age of 4 years [30]. One year later, at age of 5 years, animals of group B were fed on a single occasion 5 g BSE-infected bovine brain homogenate each [13]. Six months later, macaques from group C were orally exposed to 16 g BSE on a single occasion (C1–C3) or were exposed to BSE on multiple occasions thereby receiving a cumulative oral dose of 8, 10, and 16 g (C4, C5 and C6, respectively) as described [13]. Group K served as uninfected age- and sex-matched control for groups A, B and C (Table 1). To determine time dependent spread of prions after oral infection during the preclinical phase of infection, 4 male animals (Group D) were orally exposed to 5 g BSE each on a single occasion and euthanized 1 (n = 2) and 3 (n = 2) years post infection to trace PrP^res^ accumulations in peripheral tissues. For the present study, body weight, plasma samples, and post-mortem tissues were analyzed. All macaques were kept under biosafety level 3 (BSL3) housing conditions at the Paul-Ehrlich-Institute primate center. The females were kept in groups of six animals with a high-caloric *ad libitum* feeding.

**Table 1:** Characteristics of study animals.

Animal	Sex	Age (y)	IP (y)	Ileum→rectum (GALT PrP^res^)	Pancreas PrP^res^	Adrenal gland PrP^res^	Spinal cord PrP^res^	Brain^1^ PrP^res^	T2D onset
*Group A (i.c. BSE infection, 5 mg dose, exposed on a single occasion) – Animals were euthanized after the onset of neurological signs*									
A1	F	4	4.4	+	n.d.	n.d.	+	+	n.a.
A2	F	4	6.1	+	n.d.	n.d.	+	+	n.a.
A3	F	4	5.2	+	n.d.	n.d.	+	+	n.a.
A4	F	4	5.6	+	n.d.	n.d.	+	+	n.a.
A5	F	4	2.9	+	n.d.	n.d.	+	+	n.a.
A6	F	4	4.8	+	n.d.	n.d.	+	+	n.a.
**Mean, SD**			***4.8±1.1***						
*Group B (oral BSE infection, 5 g dose, exposed on a single occasion) – Animals were euthanized after the onset of neurological signs* *									
B1	F	5	5.2	+	neg.	neg.	+C→L	+	n.a.
B2^2^ *	F	5	n.a.	+	neg.	neg.	+L	neg.	9.0
B3	F	5	4.3	+	neg.	neg.	+C→L	+	n.a.
B4	F	5	4.8	+	neg.	neg.	+C→L	+	n.a.
B5	F	5	4.6	+	neg.	neg.	+C→L	+	n.a.
B6*	F	5	n.a.	(+)	neg.	neg.	+L	neg.	10.5
**Mean, SD**			***4.7±0.4***						
*Group C (oral BSE infection, 16 g dose, exposed on a single occasion) – Animals were euthanized after the onset of neurological signs*									
C1	F	5	3.7	+	neg.	neg.	+C→L	+	n.a.
C2	F	5	5.3	+	neg.	neg.	+C→L	+	n.a.
C3	F	5	4.5	+	neg.	neg.	+C→L	+	n.a.
**Mean, SD**			**4.5±** ***0.8***				+C→L		
*Group C (oral BSE infection, cumulative dose of 8, 10 or 16 g BSE) – Animals did not develop neurological signs* *									
C4	F	5	n.a.	+/−	neg.	neg.	+L	neg.	12
C5	F	5	n.a.	+/−	neg.	neg.	+L	neg.	12
C6	F	5	n.a.	(+)	neg.	neg.	neg.	neg.	15
*Group K (MOCK brain) – noninfected age- and sex-matched controls for A, B and C*									
K1	F	n.a.	n.a.	neg.	neg.	neg.	neg.	neg.	n.a.
K2	F	n.a.	n.a.	neg.	neg.	neg.	neg.	neg.	7.6
K3	F	n.a.	n.a.	neg.	neg.	neg.	neg.	neg.	n.a.
K4	F	n.a.	n.a.	neg.	neg.	neg.	neg.	neg.	10.3
K5	F	n.a.	n.a.	neg.	neg.	neg.	neg.	neg.	n.a.
K6	F	n.a.	n.a.	neg.	neg.	neg.	neg.	neg.	n.a.
*Group D (oral BSE infection, 5 g dose) – Animals were euthanized during the preclinical phase*									
D1	M	5	1	+	neg.	neg.	neg.	neg.	n.a.
D2	M	5	1	+/−	neg.	+/−	neg.	neg.	n.a.
D3	M	5	3	(+)	neg.	neg.	+L	neg.	n.a.
D4	M	5	3	+	neg.	neg.	+C_5–7_, +L	neg.	n.a.

Abbreviations: F, female; IP, incubation period; M, male; n.a., not applicable; n.d., not determined; neg., negative test result; y, years.

+, positive; (+), weakly positive; +/−, indeterminate results; +C→L, PrP^res^ accumulations in all spinal cord segments (cervical to lumbar); C, cervical spinal cord segments; L, lumbar spinal cord segments.

1 All parts of the brain including hypophysis and medulla oblongata.

2 T2D pathogenesis was published elsewhere [32].

* B2, B6, C4, C5, and C6 had to be euthanized for humane reasons due to severe T2D before the prion disease onset.

Blood samples were collected from anaesthetized (intramuscular injection of 2.5 mg xylazine/HCl kg-1 and 6 mg ketamine/HCl kg-1) fasted monkeys in the morning (7–8 a.m.) and stored as citrate plasma. Clinical checkups included measurements of body weight, body temperature and sporadic glucose tests using freshly obtained capillary blood.

One of the orally dosed macaques of group B presented with severe spontaneous diabetes with unique pathological features four years post infection: severe islet cell degeneration in complete absence of islet amyloid polypeptide deposits as reported recently [32].

All analyses described below were performed in the BSL3 prion laboratory unit of the Paul-Ehrlich-Institut.

Longitudinal analyses of glucose, insulin, pro-insulin, C-peptide, and glucagon concentrations were performed using archived plasma samples. Due to the lack of corresponding samples for some orally-infected and MOCK animals measurements were performed in a subset of 4 and 5 animals, respectively. Human proinsulin, ultrasensitive insulin and ultrasensitive C-peptide were measured by ELISA (Mercodia). Glucagon was also measured by ELISA (Phoenix Pharmaceuticals). Samples from one animal were always tested on the same ELISA plate to account for potential inter-assay variation. The absorbance (OD_450_) was measured using a VICTOR microtiter plate reader (PerkinElmer).

Glucose concentrations were measured in plasma samples using an ACCU-Check Aviva glucometer (Roche Diagnostics).

Post mortem samples of tissues including central and peripheral nervous system, gut, pancreas, adrenal glands, and fat were collected immediately and either fixed in 4% (w/v) buffered formalin (Roti-Histofix, pH 7, Carl Roth), in Carnoy's fixative, Bouin's fixative or stored at −80°C as described [13], [30]. Routine histopathological examinations of the brains were performed to detect spongiform brain lesions in hematoxylin and eosin (H&E)-stained sections. Paraffin-embedded tissue (*PET*) blots using anti-PrP monoclonal antibody (mAb) 12F10 (SPI-Bio/IBL Int.) and Western immunoblots using anti-PrP mAbs 6H4 (Prionics), 12F10 and 3F4 (Chemicon International) were performed as previously described [13], [30] to detect PrP^res^ accumulation in tissues and homogenized samples, respectively.

Immunostaining to detect L-cells was performed using the mouse anti-GLP1 [8G9] antibody (Abcam, Cambridge, England). Briefly, endogenous peroxidase was blocked with H_2_0_2_ (3% v/v). Tissue sections of distal ileum were then treated with Target Retrieval Solution according to the manufacturer's guidelines (DAKO Deutschland GmbH, Hamburg, Germany) and subsequently blocked with Slimfast (Allpharm Vertriebs GmbH, Messel, Germany, PZN no. 02418318). Section were incubated with primary antibody (1∶2000) at room temperature for 90 min, followed by incubation with DAKO LSAB+ System-HRP kit (horseradish peroxidase labeling), and DAKO AEC+ High Sensitivity Subtrate Chromogen (staining). Tissue sections were finally counterstained with hematoxylin.

Data are expressed as mean ± SEM. Mann-Whitney *U*-test was used for comparisons between groups and Wilcoxon signed-rank test for comparisons within a group. Simple linear regression analyses were used to determine weight, hormone, and glucose changes during the first 1.5 years. All analyses were performed using SPSS statistics software (v. 21). Statistical significance (*P*<0.05) was calculated using two-tailed tests.

## Results

The outcome of the BSE infections has been previously described [13], [30] and is summarized in Table 1. Briefly, 6/6 i.c. BSE infected macaques developed neurological signs 2.9–6.0 years post inoculation (mean incubation period 4.8 years). Post mortem examinations confirmed spongiform lesions and PrP^res^ deposits in the brain of all six animals [13], [30]. The median incubation time in orally BSE-infected macaques of group B was 4.7 years (4.3–5.2 years). Post mortem examinations confirmed spongiform changes and PrP^res^ deposits in the brain of all macaques showing neurological signs (4/6) as described [13]. All three macaques of group C that received 16 g BSE on a single occasion (C1–C3) developed neurological signs 3.7–5.3 years p.i., whereas the remaining macaques (C4–C6) that were orally exposed with BSE on multiple occasions (cumulative BSE dose 8–16 g) remained asymptomatic during the observation period as described earlier [13].

Macaques B2 and B6 developed severe diabetes and were euthanized at the age of 9 and 10.5 years, respectively, for humane reasons. Post mortem examination detected PrP^res^ deposits in spinal cord segments (Table 1), confirming that both animals were successfully infected with BSE. However, two out of six animals from group K (K2 and K4) also developed severe T2D and had to be euthanized. Macaque K2 was euthanized at the age of 7.6 years and macaque K4 at the age of 10.3 years (Table 1).

**Figure 1:**
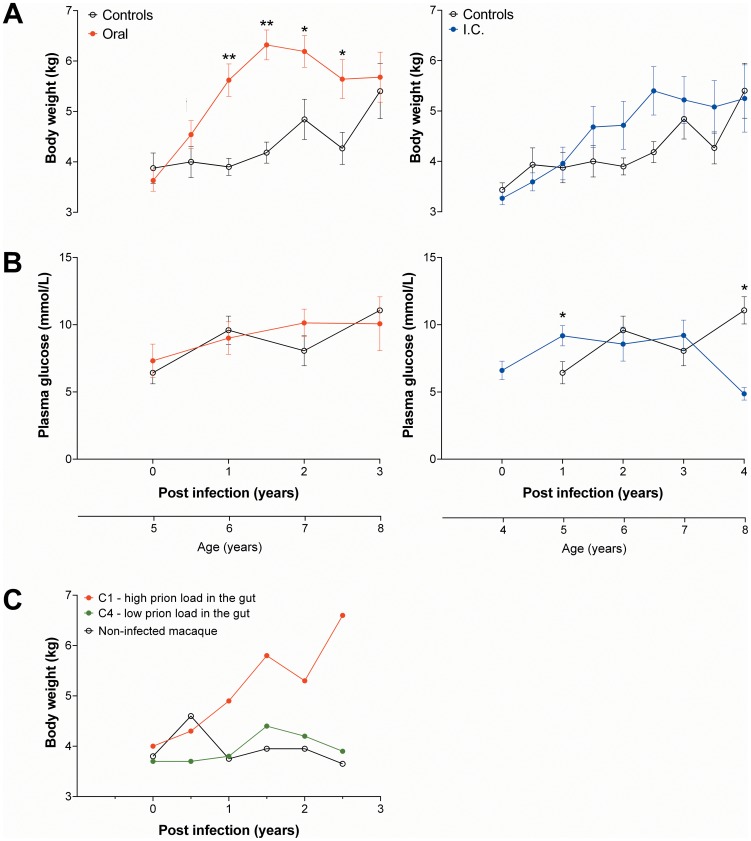
Longitudinal changes of body weight (A and C) and plasma glucose concentration (B) after BSE infection. Depicted are the body weights (A) and plasma glucose (B) of orally (n = 5, group B) and i.c. (n = 6, group A) infected animals compared with MOCK controls (n = 5–6, group K). The same MOCK group was used for comparisons with the oral and i.c. group. (C) Individual longitudinal body weights of two orally BSE-infected macaques of group C (C1 and C4) and a MOCK control animal. * - *p*<0.05, ** - *p*<0.001 († - *p*<0.05, compared to baseline of the orally-infected group; Wilcoxon signed-rank test).

In group B, oral infection with BSE resulted in weight gain within six months (Figure 1A). Although not significant compared to control group K six months after infection, the body weight of the orally infected group increased compared with baseline (*P*<0.05; Wilcoxon signed-rank test). Body weight of the orally infected animals at one year post-infection was higher compared with control animals (*P*<0.01). After 1.5 years post infection body weight reached the maximum in the orally-infected group and remained higher until 2.5 years post infection compared with the control group. Within 1.5 years post infection the body weight of the orally-infected group increased by about 80% (β = 0.915; *P*<0.0001), while the control group (β = 0.149; *P* = 0.142) did not show an increase during this time period. The body weight of the i.c.-infected group was similar to the control group (Figure 1A).

**Figure 2:**
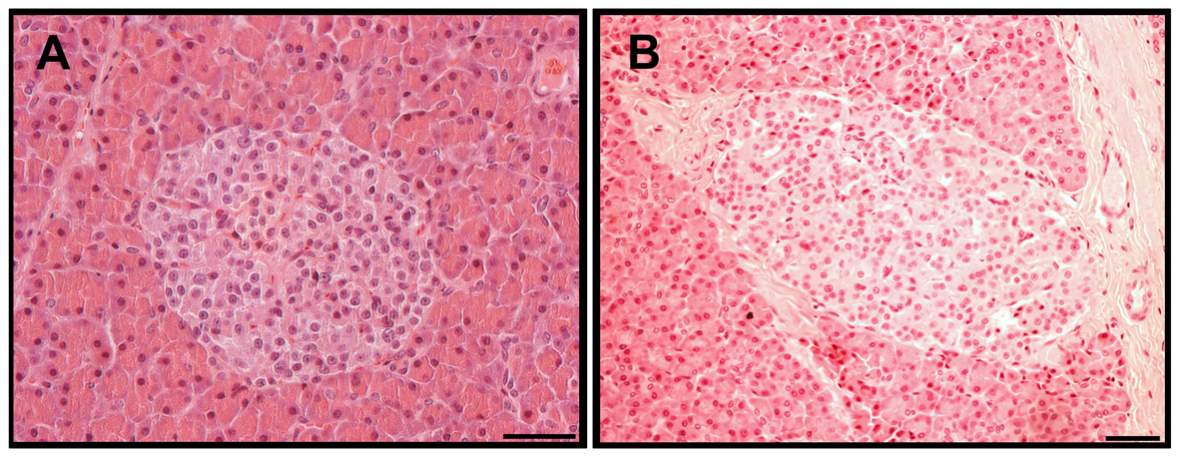
Islet morphology of BSE-infected and non-infected macaques. The morphology of pancreatic islets from a non-infected age- and sex-matched control animal (A) and an orally BSE-infected (B) macaque euthanized 4 years p.i. was similar. No PrPres was detected in islets of BSE infected monkeys (data not shown). Bars = 50 µm.

Plasma glucose concentrations in orally-infected animals (Figure 1B) remained constant during the infection period and were similar to those of the control group. In the i.c.-infected group, the plasma glucose concentrations were higher compared with controls at one year post infection and markedly lower at four years post infection (Figure 1B). Insulin, proinsulin, C-peptide, and glucagon concentrations of the orally- and i.c.-infected animals also remained stable during the infection period and were similar to the control group (data not shown). In addition, the morphology of islets of BSE-infected animals was similar to control animals (Figure 2) and PrP^res^ could not be detected in the pancreas from both preclinical and clinical cases of BSE-infected animals (Table 1).

In group C, oral infection of macaques C1–C3 also resulted in an early weight gain. However, this weight gain was not seen in macaques C4–C6 that remained clinically asymptomatic. Individual body weight change of macaques C1 and C4 is shown in Figure 1C. Macaque C1 rapidly progressed towards the clinical phase of BSE infection and the animal was euthanized at a time point when it was still gaining weight. All other macaques of groups B and C showing early weight gain post infection were euthanized years after reaching the maximum of weight.

**Figure 3:**
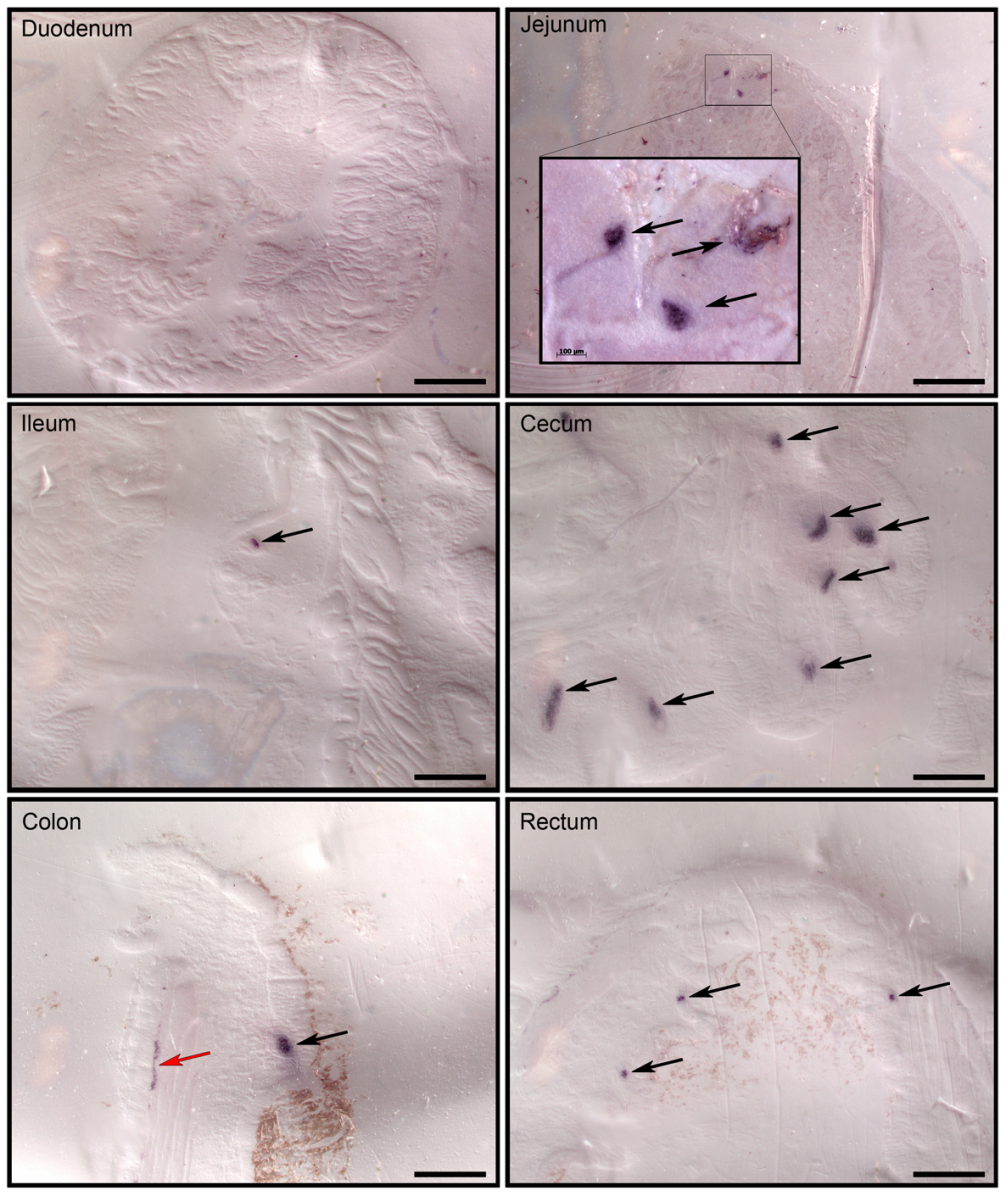
Detection of PrP^res^ deposition within the small and large intestine (transverse sections) of a clinical case using PET blot. Representative PET-blot results from macaque B3 that was euthanized 4.3 years after oral BSE uptake are shown. PrP^res^ was detected in the jejunum, in germinal centres of lymphoid follicles in the distal part of the ileum and the large intestine (black arrows). PrP^res^ deposits were also present in the gut autonomic nerve system (red arrow). Bars = 1000 µm (Inset, bar = 100 µm).

Post mortem examinations of orally BSE-infected preclinical male macaques that were euthanized 1 and 3 years post exposure revealed that prions primarily accumulated in the gut (Table 1, animals D1–D4). Consecutive serial tissue sections stained with hematoxylin and eosin (H.E.) and for PrP^res^ (*PET* blots) showed that prions had been deposited in a few germinal centers of submucosal lymphoid follicles of distal ileum and cecum. In clinical cases, prion load in the gut-associated lymphoid tissue (GALT) system increased compared with preclinical cases and included PrP^res^ deposits in germinal centers of follicles in Peyer's patches (PP), ileum-draining lymph nodes, and submucosal lymphoid follicles in cecum, the entire colon and rectum. PrP^res^ deposits in ganglia of the myenteric plexus were also detectable (Figure 3, Table 1). However, intestinal prion load in macaques C4–C6 was extremely low (Table 1). We suggested that that this outcome was at least partially caused by differences in the BSE challenge mode as described previously [13].

**Figure 4:**
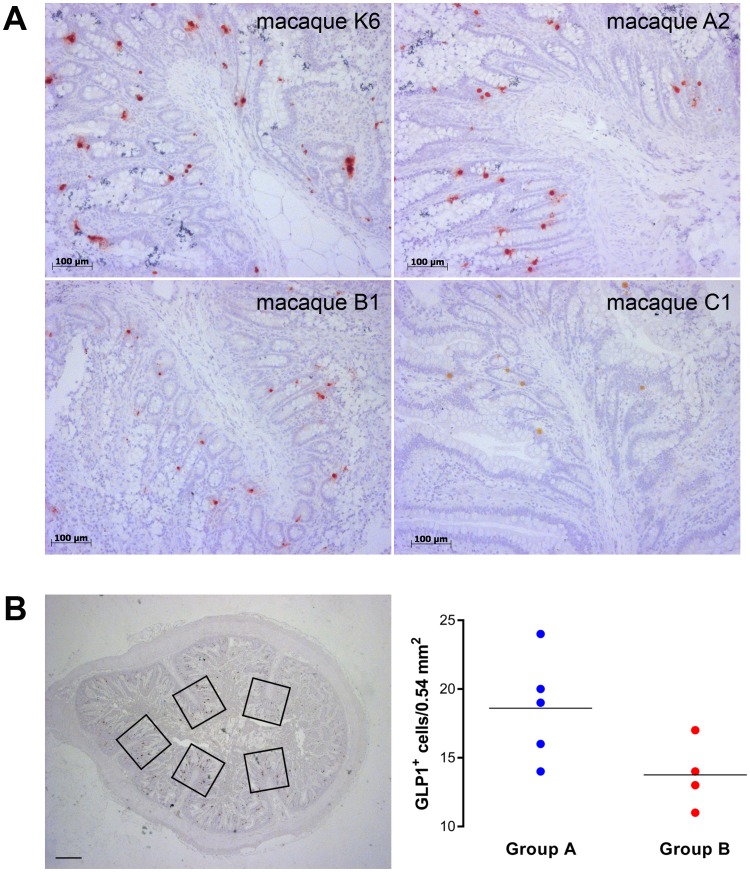
Detection and quantification of GLP-1^+^ cells in the distal ileum of BSE-infected and non-infected macaques. (**A**) Gut sections of both non-infected (representative image; animal K6) and i.c.-infected (representative image; animal A2) macaques showed similar numbers of GLP-1^+^ cells. In contrast, orally BSE-infected macaques (representative images; animal B1 and C1) showed a lower number of GLP-1^+^ cells. (**B**) GLP-1^+^ cell densities were determined by quantification of 4–8 different segments of the ileum (Bar = 1000 µm). All *Plicae circulares* in a segment were analyzed (five frames in the representative image). The total analyzed area ranged from 8.1 to 22.1 mm^2^ (17–41 frames for each animal; one frame = 0.54 mm^2^). The dot plot shows the results of the GLP-1^+^ cells quantification of four animals from the group A (i.c.-infected) and four animals from group B (orally-infected). Expressed are the means of the cell densities.

In a pilot study, we detected a reduced GLP-1^+^ cell density in the distal ileum of orally infected animals (group B) compared with intracerebrally BSE-inoculated macaques (Figure 4). Intriguingly, a strongly reduced GLP-1^+^ cell density was detected in macaque C1 (Figure 4A) that rapidly progressed to the clinical phase of BSE-infection and showed an extremely high intestinal prion load at post mortem examination. Case C4 that remained asymptomatic throughout the entire observation period exhibited an extremely low intestinal prion load in distal ileum and cecum and high GLP-1^+^ cell counts (data not shown).

## Discussion

The European study ‘BSE-in-primates’ was designed to assess the risk of humans to become infected by prion-contaminated food or blood products and to identify early blood-based changes or surrogate markers in infected macaques [13], [30]. A surprising additional finding of the study was the dramatic body weight increase within the first 1.5 years in orally but not in intracerebrally or non-infected animals. The phenomenon was seen in two animal groups (B and C) which were orally infected with BSE at two different time points six months apart. The rapid-onset obesity was not associated with abnormal plasma glucose or islet hormone concentrations. There was also no evidence for prion replication in the brain of preclinical macaques up to 3 years after foodborne BSE transmission. These observations do not favor a primary pancreas- or CNS-based mechanism of triggering the rapid weight gain after dietary BSE exposure.

At the end of the risk assessment study, we analyzed clinical and laboratory findings in BSE-infected macaques of group B (5 g single dose). All six animals developed a vCJD-like disease after approximately 5 years of incubation time. The most striking finding during the asymptomatic phase of BSE infection was a significant weight gain in macaques B1–B6 compared with non-infected age- and sex-matched controls K1–K6. However, only 3 out of 6 animals of group C (macaques C1–C3) showed weight gain within the first years post infection. These animals were exposed on a single occasion to 16 g BSE each and subsequently developed neurological signs. Post mortem examination confirmed spongiform encephalopathy and high intestinal PrP^res^ loads in all three animals. The remaining three macaques (C4–C6) were exposed on multiple occasions to BSE. This successive BSE challenge mode did not lead to spongiform encephalopathy or to a high intestinal prion load. The underlying mechanism is not known as discussed earlier [13]. However, these three animals (C4–C6) did not show any significant weight gain during the early phase after infection.

We demonstrated previously that BSE prions entered the CNS primarily at the lumbar spinal cord level most likely via lesser and lumbar splanchnic nerves 3 years post infection [13], bypassing pancreas and adrenal glands as shown in this study (Table 1). It is well known that foodborne transmitted prions replicate first in the GALT system and centripetally enter the central nervous system (CNS) via peripheral nerves [33], [34]. PrP^res^ accumulations could be detected in the distal ileum and cecum of macaques euthanized 1 year post infection. All other tissues and organs in the body of macaques did not show any evidence for prion infection at this very early time point after oral BSE uptake. However, PrP^res^ deposits were irregularly distributed in ileum and large intestine both in preclinical and clinical cases.

The coincidence between rapid weight gain and a relatively high intestinal prion load within the first 2 years post infection then suggested that the underlying mechanism for rapid onset obesity may be found in the gut. GI tract hormones called incretins are known to influence brain functions associated with the energy balance [35]–[38]. For example, GLP-1 and PYY can act as potent appetite suppressing hormones in humans and cynomolgus monkeys [39]. Moreover, GLP-1-secreting L-cells are predominantly found in ileum and colon [40], [41] which are also the main target sites for prion replication (Table 1 and [13]). We indeed observed in a pilot study a reduced GLP-1^+^ cell density in distal ileum specimens from orally BSE-infected macaques of group B compared with intracerebrally BSE-infected macaques of group A (Figure 4). In animals of group B, intestinal areas with reduced GLP-1^+^ cell density alternated with segments exhibiting high GLP-1^+^ cell numbers, whereas in animals of groups A and K, consistently high GLP-1^+^ cell density was detected in all examined specimens. The irregular prion distribution in ileum and large intestine of orally BSE-infected macaques appeared to correlate with reduced GLP-1^+^ cell density. Furthermore, the finding of higher GLP-1^+^ cell density in BSE-infected macaques with undetectable (group A) or low (animals C4–C6) intestinal PrP^res^ levels suggest a causal relation between prions and GLP-1 expression patterns. Both findings and a possible causal relationship have yet to be confirmed by additional studies. But it was striking to see that the number of *Plicae circulares* tended to be lower in orally BSE-infected macaques compared with both intracerebrally infected and non-infected macaques (data not shown), perhaps as a result of prion infection of the myenteric plexus (Figure 3). The enteric nervous system (ENS) exerts local control over mixing and propulsive movements in the intestine and this may also affect the L-cell renewal rate. In this view, prion infection of the ENS resembles typical pathologic changes observed in Parkinson's disease patients [42].

Unfortunately, the study was not originally aimed at the examination of metabolic parameters. The original study design aimed to determine the minimal infectious and lethal dose. Consequently, we did not document food consumption and did not collect adequate serum samples to determine systemic concentrations of gut endocrine hormones during the period of weight gain. The orally BSE-infected animals were euthanized years after reaching maximum weight. Moreover, the macaques showed a weight loss over a 2 to 3 year period before showing clinical disease signs. We did also not document food consumption and can therefore not state whether the observed weight gain was a result of excessive food consumption.

The major advantage of the present study is that humans and monkeys are behaviorally, physiologically, and developmentally closely related. Therefore, the observations of prion-induced obesity in macaques could translate to humans. Rapid-onset obesity has not been described so far in vCJD patients. However, it is unlikely that medical history was available from vCJD patients ≥10 years before onset of clinical signs. Unfortunately our original study design did not include assessment of plasma-derived GI tract endocrine hormones (*e.g.* GLP-1, PYY). Therefore, plasma samples were not collected adequately to measure gut hormones during the phase of weight gain. The same is true for the signaling molecules secreted by the adipose tissue (*e.g.* leptin, adiponectin). We are therefore in the process to establish a comprehensive morphometric study to quantify incretin-secreting cells in different segments of small and large intestine to characterize the density of GI hormone-secreting cells in a large number of BSE-infected and non-infected macaques.

In conclusion, oral BSE infection of macaques resulted in rapid-onset obesity in preclinical cases. This phenomenon was neither associated with pancreatic dysfunction nor with brain damage. It remains enigmatic which pathological processes and molecular mechanisms are responsible for the induction of the rapid weight gain after oral prion exposure. Histopathological and preliminary immunohistochemical analyses indicate a GI tract based pathophysiological mechanism that impacts the gut-brain axis and triggers rapid-onset obesity. Further studies, specifically designed to investigate metabolic parameters including GLP-1 and PYY and possible role of enteric nervous system are needed to characterize the impact of orally-transmitted prions on the endocrine system. In conclusion, the present findings suggest an important role of the GI tract in the development of metabolic dysfunction after dietary exposure to pathogens and demonstrate a novel role of infectious prions in the development of obesity.
